# Supplementary material for: M6A Demethylase Inhibits Osteogenesis of Dental Follicle Stem Cells via Regulating miR-7974/FKBP15 Pathway
Source: Int J Mol Sci. 2023 Nov 9;24(22):16121. doi: 10.3390/ijms242216121 (PMC10671807; doi:10.3390/ijms242216121)
Supplement: Supplementary file 1 [file ijms-24-16121-s001.zip › ijms-2684566-supplementary.pdf]

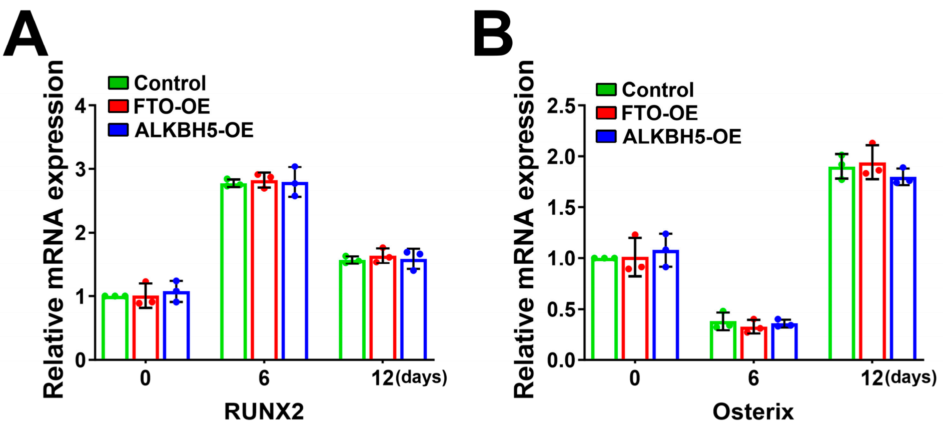


**Figure S1.** Expression patterns of RUNX2 and Osterix in FTO-OE and ALKBH5-OE DFSCs. Expression pattern of RUNX2 **(A)** or Osterix **(B)** was not obviously influenced during the 12 days' osteogenesis induction in either FTO-OE or ALKBH5-OE DFSCs. n = 3.

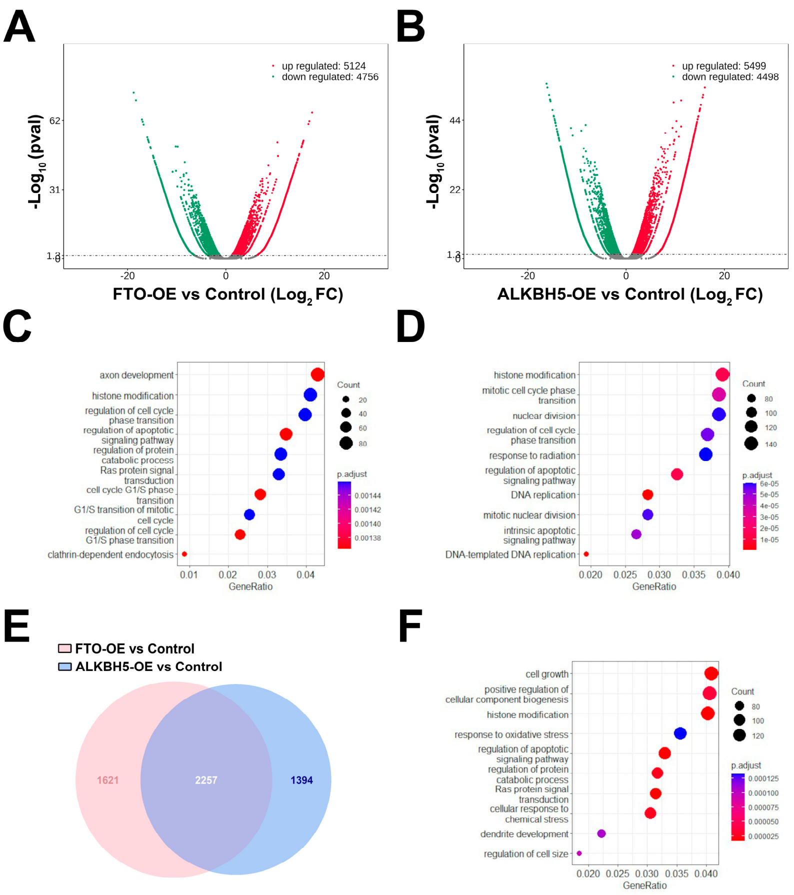


**Figure S2.** RNA sequencing of FTO-OE and ALKBH5-OE DFSCs versus vehicle control. **(A)** Volcano plot of the $Log_2$ (fold change) between FTO-OE and vehicle control and the negative $Log_{10}$ (p-value). **(B)** Volcano plot of the $Log_2$ (fold change) between ALKBH5-OE and vehicle control and the negative $Log_{10}$ (p-value). **(C)** Gene ontology classification and enrichment analysis of differentially expressed genes in FTO-OE DFSCs. **(D)** Gene ontology classification and enrichment analysis of differentially expressed genes in ALKBH5-OE DFSCs. **(E)** A total of 2257 genes were identified in the intersection of differentially expressed genes between FTO-OE cells and ALKBH5-OE DFSCs. **(F)** Gene ontology classification and enrichment analysis of commonly altered genes in FTO-OE and ALKBH5-OE DFSCs.
